# Isobaric tag for relative and absolute quantitation based quantitative proteomics reveals unique urinary protein profiles in patients with preeclampsia

**DOI:** 10.1111/jcmm.14459

**Published:** 2019-06-24

**Authors:** Wenyan Ding, Bintao Qiu, David S. Cram, Xiuting Chen, Shengjie Li, Xiya Zhou, Juntao Liu, Zhihong Wu, Yijun Song

**Affiliations:** ^1^ Department of Obstetrics and Gynecology, Peking Union Medical College Hospital Peking Union Medical College and Chinese Academy of Medical Sciences Beijing China; ^2^ Department of Central Laboratory, Peking Union Medical College Hospital Peking Union Medical College and Chinese Academy of Medical Sciences Beijing China; ^3^ Berry Genomics Corporation Beijing China

**Keywords:** differential expression, GO pathways, iTRAQ, KEGG pathways, PE, proteomics, urine

## Abstract

Preeclampsia (PE) is one of the most significant pregnancy‐related hypertensive disorders. Currently, there are no useful markers to predict the onset of the condition in pregnant women. To provide further insights into the pathogenesis of PE and identify biomarkers of the condition, we used isobaric tags for relative and absolute quantitation (iTRAQ) proteomics coupled with 2‐D LC‐MS/MS, to analyze urinary protein profiles from 7 PE patients and 7 normotensive pregnant women. A total of 294 proteins were abnormally expressed in PE patients. Of these, 233 were significantly down‐regulated and 61 proteins were significantly up‐regulated. Bioinformatics analysis using the Gene Ontology (GO) and Kyoto Encyclopedia of Genes and Genomes (KEGG) database, found that the most differentially expressed proteins (DEPs) were involved in coagulation and complement pathways, the renin‐angiotensin system and cell adhesion molecules (CAMs) pathways. We further validated three of the DEPs, including serotransferrin (TF) and complement factor B (CFB) by immunoblottingand serum paraoxonase/arylesterase 1 (PON1) by ELISA using 14 pairs of urine samples from PE patients and normal pregnant women. Taken together, our results provide the basis for further understanding the pathogenesis of PE and identifying predictive biomarkers.

## 1 INTRODUCTION

Preeclampsia (PE) is a pregnancy‐specific syndrome characterized by hypertension and proteinuria^1^ and is one of the major causes of maternal and perinatal mortality and morbidity worldwide, affecting 2%–5% of all pregnancies.^2^ PE contributes to around 20% of pregnancy‐related maternal deaths, and there is no effective treatment.^3^ PE is defined as new‐onset elevated blood pressure accompanied by proteinuria after 20 weeks of gestation.^4^ Despite extensive research, the pathogenesis of PE has not been fully unraveled. Nevertheless, it is widely accepted that PE is initiated by disturbances at the foetal‐maternal interface, followed by generalized inflammation and progressive endothelial damage.^5^

For patients with severe PE, the most effective treatment to relieve symptoms is early delivery. Several maternal risk factors such as previous history of PE with antiphospholipids antibodies, pre‐existing diabetes, multiple (twin) pregnancy, nulliparity and family history have been associated with PE.^6^ Nonetheless, it still remains extraordinarily difficult for clinicians to predict patients who will develop the condition in pregnancy. Current research has focused on mapping the changes in the levels of soluble blood vascular factors in response to disease activity.^7^, ^8^ So far, several studies have shown that angiogenic factors such as placental growth factor, serum FMS‐like tyrosine kinase‐1 and endoglin are candidate markers which appear to have reasonable value in predicting early‐onset PE in some patients. Nonetheless, the discovery of other useful biomarkers to assist precise prediction, early diagnosis and treatment monitoring of PE is urgently needed.

Since renal pathogenesis is a hallmark of PE^9^ and the degree of proteinuria has a strong positive correlation with the degree of disease severity, we postulated that the analysis of urinary proteins may provide further clues to the mechanisms of pathogenesis and useful biomarkers for predicting disease onset. To test this hypothesis, we used Isobaric tag for relative and absolute quantitation (iTRAQ) proteomics coupled with 2‐D LC‐MS/MS^10^ to compare urinary protein profiles from seven PE patients (test samples) with those of seven normotensive pregnant women without any complications (control samples). Based on the American Congress of Obstetricians and Gynecologists guidelines,^11^ five patients were diagnosed as early onset PE (<34 weeks) and two patients were diagnosed with late onset PE (≥34 weeks). Where possible, patients and controls were matched for maternal and gestational age at delivery. Prior to delivery when urine samples were taken for analysis, all seven patients had progressed to severe PE.

Using the IGY 14 depletion kit (Thermos Fisher), fresh midstream urine samples were first depleted of high molecular weight proteins including immunoglobulin and albumin, which are known to interfere with iTRAQ analysis.^12^ A total of 100 μg of deleted protein extract from each sample were then trypsinized and the resulting peptides subjected to iTRAQ and MS analysis. The Protein Prophet algorithm was used to assign the probability that each peptide was related to a particular protein. Normalization of the relative peptide intensities of protein data was carried out iteratively (across samples and spectra) using ANOVA, as previously described.^13^

**Figure 1:**
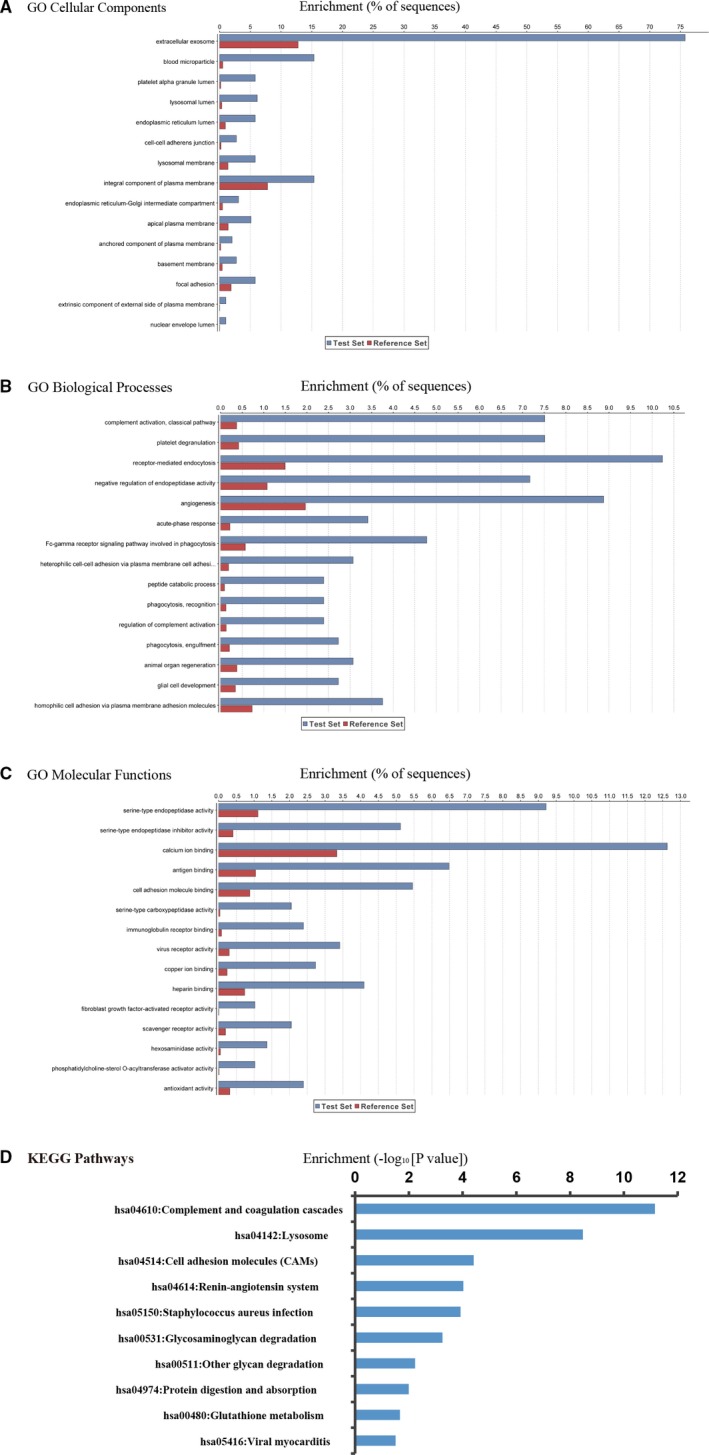
Functional analyses of preeclampsia differentially expressed proteins. Differentially expressed proteins (DEPs) enriched for different cellular components (A), biological processes (B) and molecular functions (C), according to GO annotation. KEGG pathway enrichment analysis of DEPs for significantly enriched pathways (D)

We found that the seven women with severe PE consistently showed differences in the urine proteomic fingerprint compared to their seven matching controls. Among a total of 985 proteins identified, 294 (30%) were differentially expressed proteins (DEPs) in PE patients (>1.5 fold change, *P* < 0.05). These 294 DEPs were further analysed by Blast2GO to characterize the relevant biological processes, cellular components and molecular functions associated with PE (Figure 1). We found that the vast majority of DEPs were located in the extracellular space and thus were secreted proteins (Figure 1A). Gene Ontology analysis for biological processes revealed that the main functional categories of these proteins were complement activation, platelet degranulation, receptor‐mediated endocytosis and negative regulation of endopeptidase activity (Figure 1B). According to molecular function analysis, these proteins were mainly involved in serine‐type endopeptidase activity, serine‐type endopeptidase inhibitor activity and calcium ion binding (Figure 1C). KEGG pathway analysis found that the DEPs were involved in coagulation and complement pathways, the renin‐angiotensin system and cell adhesion molecule interactions (Figure 1D). These altered biological pathways in PE patients are consistent with general observations from PE blood studies^7^, ^8^, ^9^ and a previous study of urinary proteins whereby pooled PE and control samples were also compared by iTRAQ analysis.^14^

**Figure 2:**
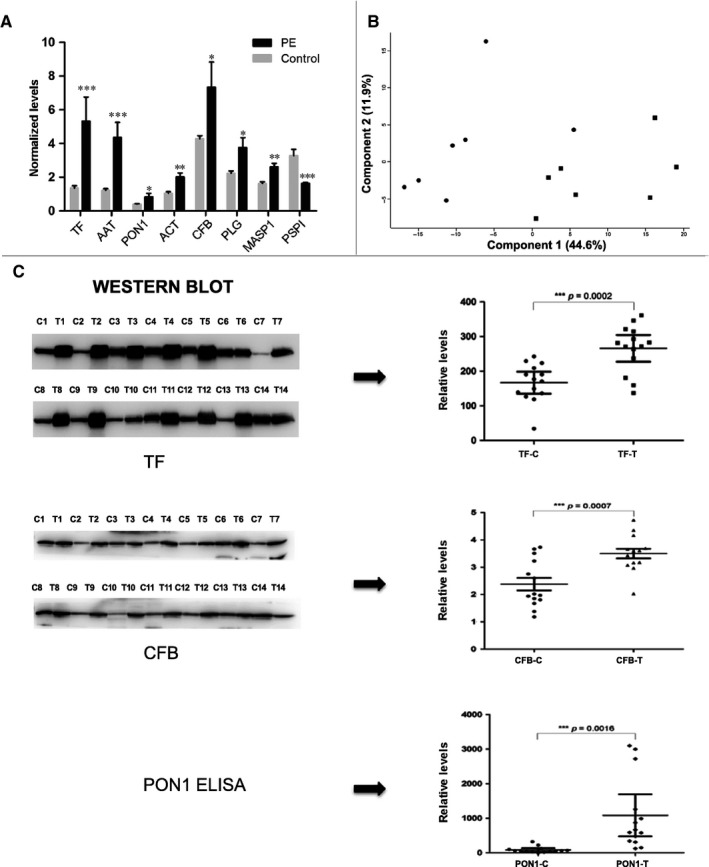
Validation of preeclampsia (PE) differentially expressed urine proteins. A, Normalized levels of selected candidate proteins Transferrin (TF), Alpha‐1‐antitrysin (AAT), Serum paraoxonase/arylestersase 1 (PON1), Alpha‐1‐antichymotrypsin (ACT), Complement factor B (CFB), Plasminogen (PLG), Mannan‐binding lectin serine protease 1 (MASP1) and plasma serine protease inhibitor (PSPI). Each bar represents the mean ± SEM. Statistical significance for the differences (*P < 0.05; **P < 0.01; ***P < 0.005) were determined by paired Student's *t* test. B, Principal components analysis. PE samples (●) and control samples (■). C, Validation of key differentially expressed proteins by Western blot or ELISA. Dot plots of the relative levels of TF, CBF and PON1 in individual test (T) group and control (C) group samples. The middle line and the top and bottom lines represent the mean ± 95%CI, based on the relative intensities of the gel bands or ELISA values. Statistical significance for the differences between the T and C groups (*P < 0.05; **P < 0.01; ***P < 0.005) was determined by Student's *t* test

Of the 294 DEPs, 233 were significantly down‐regulated in PE (Figure 2A ‐ one selected example) and 61 were significantly up‐regulated in PE (Figure 2A – seven selected examples). The top five DEPs were all up‐regulated in PE, including dynein heavy chain 8 (18.6‐fold), serum albumin (4.6‐fold, after depletion step), afamin (4.0‐fold), serotransferrin (3.9‐fold) and alpha1‐antitrypsin (3.6‐fold). The 289 remaining DEPs showed moderate fold changes ranging from as low as 1.5‐fold to as high as 3‐fold. By principal components analysis, except for one PE sample (outlier), the PE and control groups could be separated into two distinct populations (Figure 2B). In order to validate the accuracy of iTRAQ for identifying changes in levels of urine proteins in PE patients, we selected three key DEPs involved in the GO and KEGG pathways, including human serum paraoxonase/arylesterase 1 (PON1) and transferrin (TF) that was highly elevated in PE patients and complement factor B (CFB) that was only moderately elevated in PE patients (Figure 2A). Urine samples from 14 PE patients and 14 normotensive pregnant patients (including the original seven matching patients and controls) were comparatively analysed by either immunoblotting (TF and CFB) or ELISA (PON1) (Figure 2C). Based on dot plot analysis of individual samples results, the mean levels of TF (266 vs 166, *P* < 0.0002), CFB (3.5 vs 2.4, *P* < 0.0007) and PON1 (1083 vs 85, *P* < 0.0016) were significantly higher in PE patients compared to control samples. These independent assays of the three selected DEPs conducted on the original and additional urine samples confirmed the primary iTRAQ results.

This study demonstrates that iTRAQ is a powerful analytical tool for quantitating changes in levels of proteins in complex body fluids like urine. The DEPs we identified in this study represent a valuable catalogue of proteins abnormally altered at end stage PE. The challenge now is to determine whether any of these DEPs, or new unknown proteins, appear early in the urine of patients at risk for developing PE. One strategy would be to enroll pregnant patients with known risk factors for PE into a new study whereby urine samples are taken weekly from gestational age of 16 weeks before symptoms first appear^4^ and use iTRAQ proteomics to analyse significant protein changes. A larger set of normotensive pregnant patients at the same gestational age could be used as the control group. Prospective analysis would reveal any changes in the normal levels of urine proteins and therefore any significant early markers could be retrospectively identified from those patients that do develop clinical PE. By this approach, it may be possible for the first time to identify the key protein changes in urine with a strong association for development of PE, leading to the design of simple assays to identify at risk pregnancies. This would then allow careful monitoring of these pregnancies with administration of the appropriate therapies to reduce symptoms, leading to better management of a term pregnancy.

## CONFLICT OF INTEREST

DSC is an employee of Berry Genomics Corporation. The other authors confirm that there is no conflict of interest.

## AUTHOR CONTRIBUTIONS

WD and YS collected the samples, designed and conducted the experiment. WD and DSC wrote the paper. YS revised the manuscript. BQ, XC, SL and ZW analysed the data. XZ and JL provided critical review and advice.
